# Validity of Predictive Equations for Resting Energy Expenditure Developed for Obese Patients: Impact of Body Composition Method

**DOI:** 10.3390/nu10010063

**Published:** 2018-01-10

**Authors:** Najate Achamrah, Pierre Jésus, Sébastien Grigioni, Agnès Rimbert, André Petit, Pierre Déchelotte, Vanessa Folope, Moïse Coëffier

**Affiliations:** 1 Nutrition Department, Rouen University Hospital Center, 76000 Rouen, France; sebastien.grigioni@chu-rouen.fr (S.G.); agnes.rimbert@chu-rouen.fr (A.R.); andre.petit@chu-rouen.fr (A.P.); pierre.dechelotte@chu-rouen.fr (P.D.); vanessa.folope@chu-rouen.fr (V.F.); moise.coeffier@chu-rouen.fr (M.C.); 2 Normandie University, URN, INSERM UMR 1073 «Nutrition, Inflammation et Dysfonction de l’axe Intestin-Cerveau», IRIB, 76000 Rouen, France; 3 Clinical Investigation Center, CIC 1404, INSERM and Rouen University Hospital, 76000 Rouen, France; 4 Nutrition Unit, University Hospital of Limoges, 87000 Limoges, France; p.jesus@wanadoo.fr; 5 Tropical Neuroepidemiology, INSERM, U1094, 87000 Limoges, France; 6 Tropical Neuroepidemiology, Institute of Neuroepidemiology and Tropical Neurology, University Limoges, UMR_S 1094, CNRS FR 3503 GEIST, F-87000 Limoges, France

**Keywords:** resting energy expenditure, body composition, bioelectrical impedance analysis, dual-energy X-ray absorptiometry

## Abstract

Predictive equations have been specifically developed for obese patients to estimate resting energy expenditure (REE). Body composition (BC) assessment is needed for some of these equations. We assessed the impact of BC methods on the accuracy of specific predictive equations developed in obese patients. REE was measured (mREE) by indirect calorimetry and BC assessed by bioelectrical impedance analysis (BIA) and dual-energy X-ray absorptiometry (DXA). mREE, percentages of prediction accuracy (±10% of mREE) were compared. Predictive equations were studied in 2588 obese patients. Mean mREE was 1788 ± 6.3 kcal/24 h. Only the Müller (BIA) and Harris & Benedict (HB) equations provided REE with no difference from mREE. The Huang, Müller, Horie-Waitzberg, and HB formulas provided a higher accurate prediction (>60% of cases). The use of BIA provided better predictions of REE than DXA for the Huang and Müller equations. Inversely, the Horie-Waitzberg and Lazzer formulas provided a higher accuracy using DXA. Accuracy decreased when applied to patients with BMI ≥ 40, except for the Horie-Waitzberg and Lazzer (DXA) formulas. Müller equations based on BIA provided a marked improvement of REE prediction accuracy than equations not based on BC. The interest of BC to improve REE predictive equations accuracy in obese patients should be confirmed.

## 1. Introduction

Assessment of resting energy expenditure (REE) provides information for weight management and adaptation of nutritional intakes, particularly useful in obese patients [1]. Indeed, REE contributes from 50% to 75% of total energy expenditure (depending of the physical activity level) [2]. Indirect calorimetry (IC) is the non-invasive reference method to measure REE (mREE), based on the consumption of O_2_ and the production of CO_2_ [3]. However, the clinical routine use of IC is limited by the high cost of the equipment and the need of trained staff. 

Alternatively, several predictive equations have been developed to estimate REE [4]. Formulas are usually based on body weight, height, age, and sex. Moreover, several studies have assessed the validity of REE predictive equations in obese subjects with some controversial results [4,5,6,7]. Body composition (BC) assessment is needed for some of these predictive equations, to quantify fat free mass (FFM) which is an important determinant of REE [8]. However, several studies reported that including BC data does not improve the accuracy of REE predictive equations in obese patients [6,9,10,11]. These findings may be partially explained by limitations of BC measurement in the obese population.

Indeed, there are several available accurate techniques for the assessment of BC in humans [12]. The widely used method, dual-energy X-ray absorptiometry (DXA), is hardly feasible in routine clinical practice contrary to the bioelectrical impedance analysis (BIA) method [13,14]. However, several studies reported that fat mass (FM) is generally underestimated by BIA in patients with obesity [15,16,17], and thus FFM may be overestimated. The low accuracy of BIA method in obese patients may lead to underestimation or overestimation of energy needs and contributes to inadequacy in dietary prescription [9].

Previously, Laforgia et al. reported considerable differences in REE predicted from FFM assessed by two methods of BC analysis, the 2-compartment model (hydrodensitometry; hydrometry) and the 4-compartment model (hydrodensitometry; hydrometry; DXA), in 104 healthy male subjects, with body mass index (BMI) range from 18.1 to 33.4 kg/m^2^ (mean BMI, 25.2 ± 3.4). More recently, Korth et al. investigated the influence of the methodology of BC analysis on the prediction of REE in 104 healthy adults (54 women and 50 men), with BMI range from 17.6 to 40.9 kg/m^2^ (mean BMI, 25.9 ± 4.1) [11]. FFM was measured by five different methods including DXA and BIA. REE predicted from FFM by these different methods showed only small differences. However, in these two studies, authors did not evaluate the obese population specifically.

The aim of the present study was to assess the impact of BC methods, either BIA or DXA, on the accuracy of specific predictive equations developed in obese patients.

## 2. Material and Methods

### 2.1. Subjects

Patients were included at the Department of Clinical Nutrition (University Medical Center, Rouen, France) from 2010 to 2016. The inclusion criteria were: being followed for obesity (BMI ≥ 30 kg/m^2^), above the age of 18 years, and without acute diseases. After an overnight fasting period of 12 h, the same operator measured both weight and height under standardized conditions, in light clothes without shoes. BMI was calculated as body weight (kg) divided by squared height (m^2^). The study was approved by the Local Ethics Committee for Non-Interventional Studies (CERNI).

### 2.2. Indirect Calorimetry 

REE was measured by indirect calorimetry, either Deltatrac II (Datex Engström, Helsinki, Finland) or Quark RMR (Cosmed, Rome, Italy) for 30 min after a fasting period of 12 h. Previous study reported similar mean REE between Deltatrac II and QuarkRMR [18]. In twenty-four healthy subjects, REE was 1630 ± 340 kcal for DeltatracII and 1607 ± 307 kcal for QuarkRMR. A calibration with a gas of known and certified CO_2_ and O_2_ composition was completed before starting the assessment (for Deltatrac II: 4.99% CO_2_, balanced with O_2_; for Quark RMR: 5% CO_2_, 16% O_2_, balanced with nitrogen). Measurements were standardized by internal guidelines. Subjects had not been physically active before the measurement and the evening before. The subjects were in supine position and awake, with the head placed in a clear ventilated canopy. Oxygen consumption and carbon dioxide production were measured and energy expenditure was calculated by the Weir formula [19].

### 2.3. Body Composition

#### 2.3.1. Dual-Energy X-ray Absorptiometry (DXA)

DXA was performed on the whole body using a Lunar Prodigy Advance (General Electric Healthcare, Little Chalfont, UK). No specific preparation was required. All patients had their underwear on, without metal accessories worn during measurement. DXA uses an X-ray generating source, with two X-ray beams with different energy levels. FFM (lean mass and bone mineral content) and FM were assessed based on their X-ray attenuation properties.

#### 2.3.2. Bioelectrical Impedance Analysis (BIA)

FFM and FM, were determined using multifrequency BIA, Quadscan 4000 device (Bodystat Ballakaap, UK) as previously described [20] and according to the manufacturer’s recommendations. Although the Quadscan 4000 device records impedance at four frequencies (5, 50, 100, and 200 kHz), the manufacturer’s manual states that only the 50 kHz impedance is used for the calculation of total body water, on which estimations for FFM are based using proprietary equations.

### 2.4. REE Predictive Equations

The predictive equations for REE (cREE) used in our study were obtained by screening previous publications and are summarized in Table 1. We selected REE predictive equations developed for obese adult patients based or not on BC (FM and FFM) [21,22,23]. 

### 2.5. Data Analysis

Predicted REE (cREE) was compared with REE measured (mREE) by indirect calorimetry by using the paired t test. When cREE ranged between 90% to 110% of mREE, it was considered as accurate cREE (AP). cREE lower that 90% of mREE was considered as an underprediction (UP) and cREE higher that 110% of mREE as an overprediction (OP). Chi2 test was performed to compare the accuracy between equations according to BC methods and to compare the accuracy between BMI subclasses. Data were analyzed using GraphPad Prism 5.0 (GraphPad Software Inc., San Diego, CA, USA).

## 3. Results

Predictive equations were studied in 2588 obese patients with 2073 women and 515 men. The mean age was 46.2 ± 14.2 years. The mean BMI was 38.1 ± 5.3 kg·m^−2^. FFM measured by DXA and BIA was 51.3 ± 0.2 kg and 58.6 ± 0.2 kg respectively. FM measured by DXA and BIA was 48.2 ± 0.2 kg and 44.8 ± 0.2 kg respectively (Table 2).

Deltatrac was used in 512 patients while QuarkRMR was used in 2076 patients. Mean mREE was 1788 ± 6.3 kcal/24 h (Table 2). Only Müller (BIA) and HB equations provided a mean cREE with no difference from mREE (Table 3). Based or not on BC (FM and FFM), the Bernstein formula provided an accurate prediction of REE in less than 20% of cases (Table 3). Inversely, in overall obese patients, Huang, Müller, Horie-Waitzberg and HB formulas provided higher accurate prediction in more than 60% of cases. The accuracy of these equations slightly decreased when applied to patients with BMI ≥ 40, except for the Horie-Waitzberg formula. Using the BIA method, Huang and Müller formulas gave 67.6% and 67.1% of accurate predictions respectively. Using the DXA method, accurate predictions decreased to 59.7% and 65.9%, respectively. Inversely, Horie-Waitzberg and Lazzer formulas provided higher accuracy using the DXA method. Indeed, the Horie-Waitzberg formula provided 61.1% and 45.9% of accurate predictions using DXA and BIA methods, respectively (*p* < 0.05). Then, the accuracy of the predictive equations slightly decreased when applied to patients with BMI ≥ 40 in overall obese patients, except for the Horie-Waitzberg and Lazzer (DXA) formulas (*p* < 0.05) (Table 3).

In overall female obese patients, the Huang (BIA) formula provided the higher accurate prediction of REE (68.3%) (Table 4). The accuracy was higher in patients with 30 ≤ BMI < 40 (69.1%) than with BMI ≥ 40 (66.8%). In overall male obese patients, the Horie-Waitzberg (DXA) formula gave the higher accuracy for REE prediction (65.8%) (Table 5). The accuracy decreased in patients with BMI ≥ 40 (62.3%) compared to patients with 30 ≤ BMI < 40 (66.8%). Surprisingly, the Lazzer formula was not accurate and always under-predicted REE in male obese patients, while its accuracy ranged from 56.8% to 67.2% in female obese patients. 

The Müller and Huang equations based on BIA provided a marked improvement of REE prediction accuracy than those equations not based on BC. Indeed, the Müller (BIA) and Huang (BIA) equations gave 67.1% and 67.6% of accuracy respectively, while Müller and Huang (without BC) gave 60.3% and 66.5% of accuracy respectively. In women obese patients, Lazzer (DXA) provided higher accuracy than Lazzer (without BC) (66.9% and 57.9%, respectively), although the difference between the two equations decreased for patients with BMI ≥ 40. Inversely, in male obese patients, Lazzer (BIA) and Lazzer (DXA) never gave an accurate prediction while Lazzer (without BC) provided 59.6% of accurate predictions, with no influence of BMI classes. Finally, Bernstein (BIA) was also more accurate than Bernstein (without BC) which provided no accurate prediction in male obese patients.

## 4. Discussion

The present study compared REE measured by IC with REE estimated from predictive equations based or not on BC (FM and FFM) and developed for obese adult patients according to the methods used for BC assessment. 

In our study, the highest accuracy was achieved by the Huang and Müller equations (almost 67% of accurate predictions) using BIA. This is in accordance with our previous study reporting that Müller equations gave the best percentage of accurate prediction (>70% of patients), especially Müller equations using BC in patients with 25 ≤ BMI ≤ 40. In patients with BMI ≥ 40, Müller equations also gave a high percentage of accurate prediction (approximately 64%) but the best accurate prediction was obtained with the Huang equation (65%–66% of patients) [5]. Our present results are also consistent with a recent study by Marra et al. reporting that the Müller (BIA) equation provided the lowest difference between predicted and measured REE both in 670 obese males (−22 kcal per day) and 1181 obese females (+47 kcal per day) [28]. Consistent with these recent results, in our study, the Müller (BIA) equation provided a mean REE with no difference compared to mREE. Likewise, the mean REE predicted by the HB equation was not different from mREE. We previously reported that the HB equation accurately predicted REE within ±10% of the mREE in 68.5% of patients with 25 ≤ BMI ≤ 40 and in 62.4% of patients with BMI ≥ 40 [5]. Then, Marra et al. reported that the Huang (BIA) equation provided an accurate prediction of REE in almost 45% of men and 40% of women, while we found 67.6% of accurate predictions in our population of obese patients. On the other hand, regardless of the method used to assess BC, the Bernstein formula did not provide accurate prediction of REE in our study. Again, this is in accordance with Marra et al. reporting the highest REE underestimation (almost 95%) with the Bernstein (BIA) formula [28].

Interestingly, in our study, the accuracy of predictive equations slightly decreased when applied to patients with BMI ≥ 40, except for Horie-Waitzberg anad Lazzer (DXA) formulas. Previously, Marra et al. also reported that the accuracy was very low (almost 55%) for all BIA based-predictive equations used to predicted REE, particularly when the BMI was high. Indeed, in a severely obese population (BMI ≥ 40 kg/m^2^), excess of FM is associated with low REE predictive accuracy [29,30]. This can be explained by the variability in the distribution of FM (central and/or peripheral, android or gynoid) in obese patients and especially severely obese patients. Furthermore, limitations of BIA in obese patients may also be explained by inadequate BIA equations developed in normal-weight subjects, and also by hydration variability [31,32]. BIA equations developed in obese subjects should be used to enhance the accuracy of the REE equations in this population [33,34]. However, surprisingly, although DXA is considered to be the widely used reference method for body composition assessment [13], in our population, the use of the BIA method in Huang and Müller equations provided better predictions of REE. Taken together, these results suggest that the precision of the BC method seems to be of minor importance for the accuracy of the REE predictive equation in obese patients. Furthermore, most studies have shown that BC-based equations did not provide a better accuracy compared to equations using age, height, and weight in obese populations [6,9,10,11,28]. However, in the present study, Müller equations based on BIA provided a marked improvement of REE prediction accuracy than equations not based on BC (67.1% versus 60.3%). Moreover, Lazzer (DXA) also provided higher accuracy than Lazzer (without BC) (66.9% versus 57.9% respectively), in women obese patients, while Lazzer (BIA) provided 64.4% of accuracy. Previously, Johnstone et al. reported that adding BC data improved the Schofield equation’s precision in 39 obese men (30 ≤ BMI < 40) [35]. However, in this study, BC was assessed using the air-displacement plethysmography method (Bodpod). Interestingly, the authors also showed that in the absence of BC data (FM and FFM), the use of anthropometric data (waist and hip circumference, mid-upper arm circumference) provided a useful alternative methodology to improve the predictability of the Schofield equation in obese men. The interest of knowing FM and FFM body distribution to improve the REE predictive equations accuracy in obese patients should be confirmed. Moreover, formulas requiring BC have been initially developed and validated with different BC techniques. The use of other methods of BC measurement can therefore modify the prediction of the formulas and alter their validity. Indeed, the Bernstein formula validated with a measurement of BC by analysis of potassium and labelled water was tested in two studies by BIA [5,28] and in another study by plethysmography [36].

Interestingly, we observed sex differences in the accuracy of predictive equations. Indeed, the Huang (BIA) formula provided a higher accurate prediction of REE in female obese patients (68.3%), while the Horie Waitzberg (DXA) formula gave a higher accuracy for REE prediction in male obese patients (65.8%). Morever, the Lazzer formula always under-predicted REE in male obese patients, while its accuracy ranged from 56.8% to 67.2% in female obese patients. Recently, Marra et al. also reported sex differences in the prediction of REE, showing very low accuracy of predictive equations in obese women, except with the Müller (BIA) equation [28]. Sex differences in the measurement of body composition by DXA and BIA have been poorly studied. However, recent data reported no effect of sex on total body water measurement by the BIA method in healthy subjects [37] and in hemodialysis patients [38].

## 5. Strengths and Limitations

To our knowledge, this is the largest retrospective study assessing the impact of BC methods on the accuracy of the specific predictive equations developed in obese patients, in outpatients followed in a Nutrition Unit. A first limitation of our study is that the patients were mainly women (80% of the total population). However, female and male obese patients were evaluated separately in statistical analysis and we highlighted sex differences. Then, the proprietary manufacturer’s equation of the BIA device (Bodystat Quadscan 4000) is unknown and probably not adapted to each BMI class and each sex. Finally, comparison of BC assessment by DXA and BIA in obese patients has been poorly documented. A few studies have shown good concordance between the two methods [39,40] while many others have not [15,17,41,42,43,44,45,46]. These conflicting results may probably be due to some limiting factors including the use of different BIA devices with different manufacturer equations, a small population size, and the differences in age, ethnicity and body weights in the sample studied. Furthermore, while using BIA may improve the accuracy of some REE predictive equations, it is not the best method to determine BC in clinical practice.

## 6. Conclusions

This study demonstrates a wide variation in accuracy for REE predictive equations in an obese population according to (i) the use or not of BC; (ii) the BC method used, either DXA or BIA; (iii) the BMI class; and (iv) the sex. Interest of BC to improve the REE predictive equations accuracy in obese patients should be confirmed, particularly in severely obese patients. New tools are needed to give reliable REE predictions in these obese patients as recently experimented by Disse et al. [29] using an artificial neural network. In the meantime, the measurement of REE by IC can still be recommended for an accurate assessment of REE in this population.

## Figures and Tables

**Table 1:** Predictive equations for resting energy expenditure.

Equations	Units	Factors Used for Calculation	REE Predictive Equations
Harris Benedict 1919 [24]	kcal/day	Sex, BW (kg), HT (cm), age (year)	M: 13.7516 × BW + 5.0033 × HT − 6.755 × age + 66.473 F: 9.5634 × BW + 1.8496 × HT − 0.6756 × age + 655.0955
Bernstein et al. [21]	kcal/day	Sex, BW (kg), HT (cm), age (year)	M: 11.02 × BW + 10.23 × HT − 5.8 ×age – 1032 F: 7.48 × WT − 0.42 × HT − 3 × age + 844
Bernstein et al. (BC) [21]	kcal/day	FM and FFM (kg), age (year)	19.02 × FFM + 3.72 × FM − 1.55 × age + 236.7
Müller et al. (BMI ≥ 30 kg·m^−2^) [23]	MJ/day	Sex, BW (kg), HT (cm), age (year)	0.05 × BW + 1.103 × sex + 0.01586 × age + 2.924
Müller et al. (BMI, BC) [23]	MJ/day	Sex, FM and FFM (kg), age (year)	0.05685 × FFM + 0.04022 × FM + 0.808 × sex + 0.01402 × age + 2.818
Huang et al. [22]	kcal/day	Sex, BW (kg), HT (cm), age (year)	10.158 × WT + 3.933 × HT − 1.44 × age + 273.821 × sex + 60.655
Huang et al. (BC) [22]	kcal/day	Sex, FM and FFM (kg), age (year)	14.118 × FFM + 9.367 × FM − 1.515 × age + 220.863 × sex + 521.995
Lazzer et al. [25,26]	MJ/day	Sex, BW (kg), HT (m), age (year)	M: 0.048 × BW + 4.655 × HT − 0.020 × AGE − 3.605 F: 0.042 × BW + 3.619 × HT − 2.678
Lazzer et al. (BC) [25,26]	MJ/day	FM and FFM (kg), age(year)	M: 0.081 × FFM + 0.049 × FM − age × 0.019 − 2.194 F: 0.067 × FFM + 0.046 × FM + 1.568
Horie-Waitzberg et al. [27]	kcal/day	FFM (kg), BW (kg)	560.43 + (5.39 × BW) + (14.14 × FFM)

BC: body composition; BMI: body mass index; BW: body weight; HT: height; FFM: fat free mass; FM: fat mass; REE: resting energy expenditure; sex: males (M) = 1, female (F) = 0; WT: weight.

**Table 2:** Anthropometric data and indirect calorimetry measurements according to the BMI.

	Overall	30 ≤ BMI < 40	BMI ≥ 40
*n*	2588	1735	853
Gender (female %)	80.1	76.9	86.6
Age (year)	46.2 ± 14.2	47.0 ± 14.3	44.7 ± 13.9
Weight (kg)	103.5 ± 15.7	96.6 ± 12.6	117.4 ± 11.6
Height (cm)	164.8 ± 8.2	165.7 ± 8.3	163.0 ± 7.6
BMI (kg·m^2^)	38.1 ± 5.3	35.1 ± 2.7	44.3 ± 3.7
FFM by DXA (kg)	51.3 ± 9.8	49.8 ± 10.1	54.3 ± 8.3
FM by DXA (kg)	48.2 ± 9.8	45.4 ± 7.1	57.7 ± 7.6
FFM by BIA (kg)	58.6 ± 11.1	57.84 ± 11.6	60.3 ± 9.7
FM by BIA (kg)	44.8 ± 11.8	38.8 ± 7.4	57.1 ± 9.1
mREE (kcal/J)	1788 ± 321	1724 ± 311	1918 ± 294

Values are expressed as means ± SD; BIA: bioelectrical impedance analysis; BMI: body mass index; DXA: dual-energy X-ray absorptiometry; FFM: fat free mass; FM: fat mass; REE: resting energy expenditure; mREE: measured resting energy expenditure.

**Table 3:** Comparison of predicted REE with measured REE for obese patients.

	Overall				30 < BMI < 40				BMI > 40			
	REE	AP (%)	UP (%)	OP (%)	REE	AP (%)	UP (%)	OP (%)	REE	AP (%)	UP (%)	(OP (%)
Measured REE	1788 ± 321				1724 ± 311				1918 ± 294			
HB1919	1799 ± 258	64.5	13.6	21.9	1738 ± 255	65.4	12.9	21.7	1921 ± 219	62.7	15.2	22.1
Bernstein	1869 ± 959 *	15.6	64.1	20.3	1888 ± 820 *	15.7	60.7	23.6	1832 ± 822 *	15.6	70.9	13.5
Bernstein (DXA)	1320 ± 196 *	3.2	95.6	0.2	1273 ± 193 *	2.9	96.8	0.3	1415 ± 162 *	3.7	96.1	0.3
Bernstein (BIA)	1447 ± 218 *	15.7 ^a^	83.8	0.5	1408 ± 222 *	17.1 ^a^	82.3	0.6	1527 ± 185 *	12.8 ^a,b^	86.8	0.4
Muller	1759 ± 202 *	60.3	20.5	19.2	1674 ± 166 *	60.3	23.1	16.6	1931 ± 152 *	60.1	15.4	24.5
Muller (DXA)	1716 ± 221 *	65.9	23.5	10.6	1654 ± 214 *	66.5	23.3	10.2	1841 ± 178 *	64.7	23.9	11.4
Muller (BIA)	1784 ± 235	67.1	14.3	18.6	1718 ± 229	68.1	14.3	17.6	1916 ± 187	65.1	14.2	20.7
Lazzer	1854 ± 242 *	58.2	10.1	31.7	1797 ± 240 *	57.1	9.0	33.9	1968 ± 199 *	59.3	12.3	28.4
Lazzer (DXA)	1555 ± 324 *	53.6	33.6	12.8	1451 ± 294 *	51.7	36.4	11.9	1763 ± 280 *	57.4 ^b^	27.7	14.9
Lazzer (BIA)	1642 ± 309 *	51.6	27.0	21.4	1535 ± 278 *	50.4	30.4	19.2	1857 ± 255 *	54.0	20.4	25.6
Huang	1748 ± 241 *	66.5	19.3	14.2	1689 ± 241 *	67.4	18.7	13.9	1867 ± 194 *	64.7	20.7	14.6
Huang (DXA)	1671 ± 227 *	59.7	33.4	6.9	1612 ± 224 *	61.0	32.6	6.4	1791 ± 184 *	57.0 ^b^	34.8	8.2
Huang (BIA)	1744 ± 241 *	67.6 ^a^	19.7	12.7	1682 ± 239 *	68.7 ^a^	19.3	12.0	1870 ± 192 *	65.4 ^a^	20.3	14.3
Horie Waitzberg (DXA)	1844 ± 207 *	61.1	9.0	29.9	1785 ± 200 *	59.4	8.6	31.9	1962 ± 168 *	64.2 ^b^	9.9	25.9
Horie Waitzberg (BIA)	1947 ± 223 *	45.9 ^a^	3.5	50.6	1899 ± 223 *	41.9 ^a^	2.7	55.4	2046 ± 185 *	53.9 ^a,b^	5.4	40.7

REE, resting energy expenditure (kcal/day, mean ± SD); DXA, dual-energy X-ray absorptiometry; BIA, bioelectrical impedance analysis, BMI, body mass index (kg·m^−2^), AP, accurate prediction (%). UP, under-prediction (%), OP, over-prediction (%). * *p* < 0.05 vs. measured REE; ^a^ *p* < 0.05 vs. DXA (Chi^2^ test) and ^b^ *p* < 0.05 vs. BMI between 30 and 40 kg·m^−2^ (Chi^2^ test).

**Table 4:** Comparison of predicted REE with measured REE for female obese patients.

	Overall				30 < BMI < 40				BMI > 40			
	REE	AP (%)	UP (%)	OP (%)	REE	AP (%)	UP (%)	OP (%)	REE	AP (%)	UP (%)	(OP (%)
Measured REE	1709 ± 272				1617 ± 223				1874 ± 274			
HB1919	1706 ± 200	66.1	13.7	20.2	1629 ± 149	66.8	12.6	20.6	1845 ± 207 *	64.9	15.9	19.2
Bernstein	1393 ± 149 *	19.5	78.0	0.5	1332 ± 103 *	20.4	78.9	0.7	1502 ± 157 *	18.0	81.7	0.3
Bernstein (DXA)	1258 ± 171 *	3.1	96.7	0.2	1194 ± 129 *	2.9	96.9	0.2	1373 ± 178 *	3.4	96.5	0.1
Bernstein (BIA)	1370 ± 179 *	14.4 ^a^	85.1	0.5	1315 ± 150 *	16.1 ^a^	83.4	0.5	1468 ± 185 *	11.4 ^a,b^	88.5	0.1
Muller	1736 ± 202 *	65.5	11.2	27.3	1635 ± 149 *	67.2	11.8	21.0	1918 ± 152 *	62.5 ^b^	10.2	27.3
Muller (DXA)	1647 ± 177 *	67.3	21.7	13.0	1563 ± 132 *	68.0	21.1	10.9	1798 ± 143 *	66.2	23.0	10.8
Muller (BIA)	1709 ± 187	67.7	13.6	22.7	1621 ± 145	68.5	13.0	18.5	1868 ± 145	66.2	13.8	20.0
Lazzer	1780 ± 187 *	57.9	8.2	39.9	1701 ± 151 *	56.8	7.1	36.1	1921 ± 159 *	59.8	11.5	28.7
Lazzer (DXA)	1685 ± 189 *	66.9	17.1	19.0	1591 ± 133 *	67.2	17.4	15.4	1855 ± 154	66.3	16.5	17.2
Lazzer (BIA)	1760 ± 200 *	64.4	8.7	31.9	1661 ± 144 *	65.5	9.4	25.1	1938 ± 151 *	62.4	8.0	29.6
Huang	1662 ± 175 *	67.4	19.9	15.7	1580 ± 133 *	68.1	19.3	12.6	1821 ± 139 *	66.2	21.1	12.7
Huang (DXA)	1593 ± 169 *	59.7	33.6	7.7	1510 ± 122 *	61.3	32.7	6.0	1742 ± 137 *	56.8 ^b^	35.7	7.5
Huang (BIA)	1660 ± 177 *	68.3 ^a^	20.8	13.9	1574 ± 134 *	69.1 ^a^	19.7	11.2	1815 ± 137 *	66.8 ^a^	20.7	12.5
Horie Waitzberg (DXA)	1784 ± 172 *	59.9	6.6	40.5	1705 ± 128 *	57.3	5.8	36.9	1927 ± 145 *	64.5 ^b^	8.1	27.4
Horie Waitzberg (BIA)	1875 ± 170 *	44.1 ^a^	2.7	63.2	1806 ± 144 *	38.3 ^a^	1.6	60.1	1998 ± 142 *	54.7 ^a,b^	4.7	40.6

REE, resting energy expenditure (kcal/d, mean ± SD); DXA, dual-energy X-ray absorptiometry; BIA, bioelectrical impedance analysis, BMI, body mass index (kg·m^−2^), AP, accurate prediction (%). UP, under-prediction (%), OP, over-prediction (%). * *p* < 0.05 vs. measured REE; ^a^ *p* < 0.05 vs. DXA (Chi^2^ test) and ^b^ *p* < 0.05 vs. BMI between 30 and 40 kg·m^−2^ (Chi^2^ test).

**Table 5:** Comparison of predicted REE with measured REE for male obese patients.

	Overall				30 < BMI < 40				BMI > 40			
	REE	AP (%)	UP (%)	OP (%)	REE	AP (%)	UP (%)	OP (%)	REE	AP (%)	UP (%)	(OP (%)
Measured REE	2127 ± 328				2081 ± 297				2281 ± 377			
HB1919	2115 ± 360	57.8	13.5	28.7	2094 ± 238	60.6	14.2	25.2	2183 ± 604	48.2 ^b^	10.6	41.3
Bernstein	3706 ± 533 *	0.0	0.0	100	3723 ± 276 *	0.0	0.0	100	3647 ± 984 *	0.0	0.0	100
Bernstein (DXA)	1529 ± 252 *	3.7	95.7	0.6	1529 ± 167 *	3.0	96.5	0.5	1527 ± 426 *	6.0	93.0	0.0
Bernstein (BIA)	1712 ± 273 *	20.8 ^a^	78.1	1.1	1709 ± 173 *	20.4 ^a^	78.9	0.7	1720 ± 473 *	21.9 ^a^	75.4	1.7
Muller	1853 ± 170 *	39.0	58.2	2.8	1805 ± 152 *	37.4	60.9	1.7	2021 ± 116 *	44.7	48.7	6.6
Muller (DXA)	1995 ± 152 *	60.2	30.3	9.5	1959 ± 140 *	61.6	30.4	8.0	2121 ± 122 *	55.2	29.9	14.9
Muller (BIA)	2082 ± 161 *	64.8	18.3	16.9	2040 ± 148 *	66.8	18.8	14.4	2228 ± 111	57.9 ^b^	16.5	25.6
Lazzer	2133 ± 212	59.6	15.6	24.8	2094 ± 207	57.9	15.1	24.0	2269 ± 169	56.1	16.5	27.4
Lazzer (DXA)	1011 ± 216 *	0.0	99.8	0.2	965 ± 189 *	0.0	99.7	0.3	1171 ± 165 *	0.0	99.0	1.0
Lazzer (BIA)	1148 ± 216 *	0.0	99.0	0.0	1095 ± 201 *	0.0	100	0.0	1335 ± 153 *	0.0	99.1	0.9
Huang	2091 ± 149 *	62.7	17.1	20.2	2053 ± 138	64.8	17.0	18.2	2223 ± 103	55.3	17.4	27.3
Huang (DXA)	1987 ± 153 *	59.6	31.5	8.9	1952 ± 134 *	60.1	32.2	7.7	2110 ± 114 *	57.9	29.0	13.1
Huang (BIA)	2081 ± 153 *	64.8 ^a^	17.8	17.4	2040 ± 141 *	67.3 ^a^	18.0	14.7	2225 ± 100	56.1 ^b^	17.6	26.3
Horie Waitzberg (DXA)	2084 ± 158 *	65.8	18.7	15.5	2054 ± 153	66.8	17.7	15.5	2189 ± 131 *	62.3	21.8	15.9
Horie Waitzberg (BIA)	2241 ± 2241 *	53.0 ^a^	7.0	40.0	2208 ± 154*	54.1 ^a^	6.5	39.4	2358 ± 119 *	49.1 ^a^	8.8	42.1

REE, resting energy expenditure (kcal/day, mean ± SD); DXA, dual-0energy X-ray absorptiometry; BIA, bioelectrical impedance analysis, BMI, body mass index (kg·m^−2^), AP, accurate prediction (%). UP, under-prediction (%), OP, over-prediction (%). * *p* < 0.05 vs. measured REE; ^a^ *p* < 0.05 vs. DXA (Chi^2^ test) and ^b^ *p* < 0.05 vs. BMI between 30 and 40 kg·m^−2^ (Chi^2^ test).
